# Stereochemical preference toward oncotarget: Design, synthesis and *in vitro* anticancer evaluation of diastereomeric β-lactams

**DOI:** 10.18632/oncotarget.18077

**Published:** 2017-05-22

**Authors:** Fabián Olazarán-Santibáñez, Debasish Bandyopadhyay, Pilar Carranza-Rosales, Gildardo Rivera, Isaías Balderas-Rentería

**Affiliations:** ^1^ Department of Chemistry, The University of Texas-Rio Grande Valley, Edinburg, Texas, 78539, USA; ^2^ Universidad Autonoma de Nuevo Leon, Facultad de Ciencias Químicas, Ciudad Universitaria, San Nicolás de los Garza, Nuevo León, 64451, México; ^3^ Centro de Investigación Biomédica del Noreste, Instituto Mexicano del Seguro Social, Monterrey, Nuevo León, 64700, México; ^4^ Centro de Biotecnología Genómica, Instituto Politécnico Nacional, Reynosa, Tamaulipas, 88710, México

**Keywords:** β-Lactam, diastereoisomer, molecular docking, β-Tubulin, cancer

## Abstract

**Purpose:**

In the battle against cancer discovery of new and novel chemotherapeutic agent demands extreme obligation. Development of anticancer compounds with higher potency and reduced side-effects is timely and challenging.

**Experimental Design:**

A small series of fourteen diastereomeric β-lactams (seven pairs) were synthesized through multi-step process exploring [2+2] ketene-imine cycloaddition as the key step. Comparative stereochemical preferences were studied through computational docking and validated by *in vitro* evaluation. β-tubulin was considered as possible molecular target and *in vitro* anticancer evaluation was conducted against SiHa, B16F10, K562 and Chang cell lines. Caspase-3 activation assay and hematoxylin/eosin staining of the cells were also accomplished.

**Results:**

Better docking scores of the *cis*- over the *trans*-β-lactams indicated favorable β-lactam—β-tubulin interactions in *cis*-geometry. *In vitro* (IC_50_) evaluation confirmed better anticancer activity of the *cis*-diastereoisomers. Apoptosis-induced cell death was supported by caspase-3 activation study. A *cis*-β-lactam [(±)-*Cis*-3-amino-1-phenyl-4-(*p*-tolyl) azetidin-2-one, 6C] was found to be more active (*in vitro*) than the marketed natural drug colchicine against SiHa and B16F10 (six times higher potency) cell lines. Reduced toxicity (compared to colchicine) in Chang cells confirmed better site-selectivity (accordingly less side-effects) of 6C than colchicine. Aside from 6C, most of the reported molecules demonstrated good to strong *in vitro* anticancer activity against SiHa and B16F10 cancer cell lines.

**Conclusions:**

Stereochemical preferences of the *cis*-β-lactams over their *trans*-counterparts, toward the molecular target β-tubulin, was confirmed by docking studies and *in vitro* anticancer evaluation. Apoptosis was identified as the cause of cell death. The lead 6C exhibited higher potency and selectivity than the marketed drug colchicine both *in silico* as well as *in vitro*.

## INTRODUCTION

Cancer is one of the leading causes of death globally and is expected to increase approximately 70% in the next two decades [1]. Although cancer is not a new disease, the description of cancer is found in several ancient literatures since 3000 BC, yet it is one of the primary reasons of death till today and subsequently one of the worse enemies of modern civilization. While radiotherapy, immunotherapy and surgical treatment play significant role in the battle against cancer but chemotherapy, either by own or in combination with other therapies, is still the most widely used strategy for the treatment of cancer. Moreover, several types of cancer have been developed drug resistance for which no effective drugs are available in the market [2, 3]. Therefore, it is important to develop new and selective anticancer drugs with higher potency and reduced side effects.

Since the discovery of penicillin in 1928, the four-membered cyclic amide (frequently known as 2-azetidinone or β-lactam) possesses the central position in drug discovery research, in particular, to treat a wide range of microbial infection. Notably, the β-lactam pharmacophore is present in a huge number of molecules (both natural and synthetic) that dominate worldwide the market of antibiotics [4–6]. In contrary, many compounds (natural, synthetic or semi-synthetic) are being used in cancer chemotherapy but the use of β-lactam derivatives in cancer chemotherapy is still unknown. This might be due to the reason that the research on β-lactam as anticancer agents is comparatively new and not been broadly explored. In fact, the anticancer activity of diversely substituted *trans*-β-lactams have been reported [7–15] to some extent and after careful search of available literatures we did not find a single example targeted to comparative computational and biological studies of anticancer potential of *cis*- and *trans*-β-lactams. To the best of our knowledge, this is the first report that systematically correlates the diastereoselectivity (*cis*- and *trans*-) of β-lactams with anticancer activity.

## RESULTS

### Chemistry

**Scheme 1:**
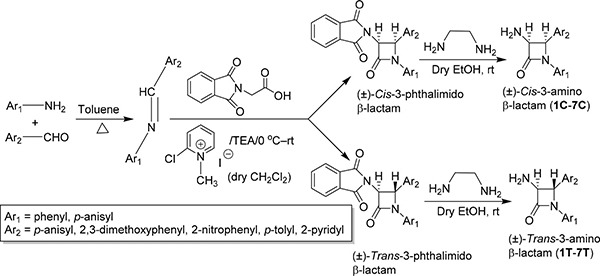
Diastereoselective synthesis of β-lactams via [2+2] ketene-imine cycloaddition reaction

In the present study Staudinger [2+2] ketene-imine cycloaddition reaction [16] was performed extensively for the diastereomeric synthesis of diversely substituted 3-phthalimido β-lactams. In the subsequent step, the deprotection of the primary amine was carried out by converting the 3-phthalimido β-lactams to 3-amino-azetidin-2-ones with ethylene diamine in dry ethanol (Scheme 1).

A total of 14 β-lactam derivatives (seven pairs of diastereomers) have been synthesized (Supplementary Table 1) through multi-step process (Scheme 1) exploring [2+2] ketene-imine cycloaddition as the key step. Although the compounds were synthesized through multi-step process and purified by repeated column chromatography yet good to excellent yields were isolated each cases. The structure and druglikeness of each molecule are shown in Table 1.

**Table 1:** Molecular docking of the β-lactam derivatives on the binding sites of the β-tubulin

Code	Docking score (kcal/mol) Colchicine site	Docking score (kcal/mol) Taxol site	Docking score (kcal/mol) Vinca site
1C	−6.6	−6.7	−6.7
2C	−7.0	−6.4	−6.1
3C	−6.8	−6.4	−6.2
4C	−7.2	−6.3	−6.8
5C	−7.2	−6.7	−6.7
**6C**	**−7.6**	−7.0	−6.9
7C	−6.5	−6.2	−6.0
1T	−6.6	−6.7	−6.6
2T	−7.2	−6.7	−6.9
3T	−6.8	−6.6	−6.6
4T	−7.3	−6.9	−6.9
5T	−7.4	−6.9	−7.3
6T	−7.4	−6.8	−7.0
7T	−6.7	−6.3	−6.5
**Colchicine**	**−7.4**	---	---
**Paclitaxel**	---	**−9.8**	---
**Vinblastine**	---	---	**−10.0**

The structure of the 2-azetidinone derivatives were elucidated by extensive spectroscopic analyses as follows:

(±)-*Cis*-3-amino-1,4-*bis*(*4*-methoxyphenyl) azetidin-2-one (1C). Brown solid (78%); Formula Weight: 298.34; MP 102 ºC; IR (KBr) 3401, 3012, 1715,1507, 1246, 826 cm^-1^; ^1^H NMR (600 MHz, CDCl_3_) δ 7.32 (d, 2H), 7.20 (d, 2H), 6.95 (d, 2H), 6.81 (d, 2H), 5.21 (d, *J* = 5.0 Hz, 1H), 4.57 (d, *J* = 5.0 Hz, 1H), 3.82 (s, 3H), 3.77 (s, 3H). Formula: C_16_H_16_N_2_O_2_; Anal. calcd for: C, 71.6%; H, 6.0%; N, 10.4%.

(±)-*Cis*-3-amino-4-(*4*-methoxyphenyl)-1-phenyl-azetidin-2-one (2C). Brown solid (88%); Formula Weight: 268.32; MP 106 ºC; IR (KBr) 3341, 2928,1728, 1499, 1248, 746 cm^-1^; ^1^H NMR (600 MHz, CDCl_3_) δ 7.29 – 7.08 (m, 2H), 7.03 – 6.93 (m, 2H), 6.86 – 6.77 (m, 4H), 4.56 (d, *J* = 2.2 Hz, 1H), 3.96 (d, *J* = 2.3 Hz, 1H), 3.71 (s, 3H) 1.93 (s,2H). Anal. calcd for: C_17_H_18_N_2_O_3_ Composition: C, 68.4%; H, 6.1%; N, 9.4%.

(±)-*Cis*-3-amino-4-(3,4-dimethoxyphenyl)-1-(4-methoxyphenyl) azetidin-2-one (3C). Brown solid (68%); Formula Weight: 328.36; MP 92 ºC; IR (KBr) 3379, 2956, 1738, 1509,1237, 828 cm^-1^; ^1^H NMR (600 MHz, CDCl_3_) δ 6.83 – 6.80 (m, 2H), 6.79 – 6.76 (m, 4H), 6.75 – 6.74 (m, 9H),, 5.10 (d, *J* = 5.4 Hz, 1H), 4.48 (d, *J* = 5.4 Hz, 1H), 3.80 - 3.68 (m, *9*H). Anal. calcd for: C_18_H_20_N_2_O_4_; Composition: C, 65.8%; H, 6.1%; N, 8.5%.

(±)-*Cis*-3-amino-1-(4-methoxyphenyl)-4-(2-nitrophenyl) azetidin-2-one (4C). Brown solid (52%); Formula Weight: 313.31; MP 185 ºC; IR (KBr) 3325, 2952, 1747, 1509, 1248, 822 cm^-1^; ^1^H NMR (600 MHz, CDCl_3_) δ 8.08 – 8.03 (m, 3H), 7.79 – 7.76 (m, 3H), 7.75 – 7.70 (m, 1H), 7.70 – 7.66 (m, 6H), 7.62 – 7.56 (m, 6H), 6.08 (d, *J* = 5.8 Hz, 5H), 6.05 (d, *J* = 5.8 Hz, 5H), 5.35 – 5.27 (m, 1H), 3.83 (s, 3H). Anal. calcd for: C_16_H_15_N_3_O_4_; Composition: C 61.3%, H 4.8%, N 13.4%.

(±)-*Cis*-3-amino-1-(4-methoxyphenyl)-4-(*p*-tolyl) azetidin-2-one (5C). Brown solid (80%); Formula Weight: 282.34; MP 103 ºC; IR (KBr)3359, 2934, 1730, 1510, 1245, 823 cm^-1^; ^1^H NMR (600 MHz, CDCl_3_) δ 7.24 – 7.20 (m, 6H), 7.14 (d, *J* = 8.1 Hz, 3H), 7.07 (d, *J* = 8.1 Hz, 1H), 6.74 – 6.70 (m, 7H), 5.13 (d, *J* = 5.4 Hz, 3H), 4.50 (d, *J* = 5.5 Hz, 3H), 3.68 (s, 3H) 2.36 – 2.32 (m, 3H). Anal. calcd for: C_17_H_18_N_2_O_2_; Composition: C, 72.3%; H, 6.4%; N, 9.9%.

(±)-*Cis*-3-amino-1-phenyl-4-(*p*-tolyl) azetidin-2-one (6C). Brown solid (84%); Formula Weight: 252.31; MP 130 ºC; IR (KBr) 3356, 2925, 1731, 1502, 748 cm^-1^; ^1^H NMR (600 MHz, CDCl_3_) δ 7.73 (dt, *J* = 13.6, 6.9 Hz, 2H), 7.71 – 7.65 (m, 2H), 7.43 – 7.35 (m, 2H), 7.26 (ddd, *J* = 8.1, 5.0, 2.4 Hz, 2H), 6.94 – 6.87 (m, 4H), 5.68 (d, *J* = 5.5 Hz, 1H), 5.46 (d, *J* = 5.5 Hz, 1H), 3.82 (s, 3H). Anal. calcd for: C_16_H_16_N_2_O; Composition: C, 76.2%; H, 6.4%; N, 11.1%.

(±)-*Cis*-3-amino-1-(4-methoxyphenyl)-4-(2-pyridyl)azetidin-2-one (7C). Brown solid (51%); Formula Weight: 269.30; MP 120 ºC; IR (KBr) 3373, 2952, 1721, 1509, 1239, 828 cm^-1^; ^1^H NMR (600 MHz, CDCl_3_) δ 8.71 (d, *J* = 4.0 Hz, 3H), 7.74 (td, *J* = 7.7, 1.6 Hz, 3H), 7.33 (d, *J* = 7.7 Hz, 4H), 7.31 – 7.27 (m, 13H), 6.82 (d, *J* = 9.1 Hz, 6H), 5.32 (d, *J* = 5.4 Hz, 3H), 4.76 (d, *J* = 5.5 Hz, 3H), 3.77 (s, 3H). Anal. calcd for: C_15_H_15_N_3_O_2_; Composition: C, 66.9%; H, 5.6%; N, 15.6%.

(±)-*Trans*-3-amino-1,4-*bis*(*4*-methoxyphenyl) azetidin-2-one (1T). Brown solid (71%); Formula Weight: 268.32; MP 96 ºC; IR (KBr) 3341, 2953, 1724,1510, 1245, 823 cm^-1^; ^1^H NMR (600 MHz, CDCl_3_) δ 7.19 (d, 2H), 7.14 (d, 2H), 6.81 (d, 2H), 6.69 (d, 2H), 4.57 (d, *J* = 1.6 Hz, 1H), 3.96 (d, *J* = 1.7 Hz, 1H), 3.72 (s, 3H), 3.66 (s, 3H). Anal. calcd for: C_16_H_16_N_2_O_2_; Composition: C, 71.6%; H, 6.0%; N, 10.4%.

(±)-*Trans*-3-amino-4-(*4*-methoxyphenyl)-1-phenyl-azetidin-2-one (2T). Brown solid (72%); Formula Weight: 298.34; MP 102 ºC; IR (KBr) 3342, 3065,1730, 1513, 1249, 747 cm^-1^; ^1^H NMR (600 MHz, CDCl_3_) δ 7.29 – 7.08 (m, 4H), 7.03 (t, 1H), 6.86 (d, 4H), 4.56 (d, *J* = 2.2 Hz, 1H), 3.96 (d, *J* = 2.3 Hz, 1H), 3.71 (s, 3H) 1.84 (s,2H). Anal. calcd for: C_17_H_18_N_2_O_3_Composition: C, 68.4%; H, 6.1%; N, 9.4%.

(±)-*Trans*-3-amino-4-(3,4-dimethoxyphenyl)-1-(*4*-methoxyphenyl) azetidin-2-one (**3T**). Brown solid (93%); Formula Weight: 328.36; MP 104 ºC; IR (KBr) 3301, 3001, 1733, 1508,1237, 877 cm^-1^; ^1^H NMR (600 MHz, CDCl_3_) δ 7.32 (d, 13H), 7.25 (d, 9H), 6.91 (d, 12H), 6.87 (d, 6H), 6.85 – 6.83 (m, 10H), 6.81 (d,13H), 6.76 (s, 1H), 4.61 (d, *J* = 1.7 Hz, 5H), 4.07 (d, *J* = 1.7 Hz, 7H), 3.89 – 3.77 (m, 9H). Anal. calcd for: C_18_H_20_N_2_O_4_; Composition: C, 65.8%; H, 6.1%; N, 8.5%.

(±)-*Trans*-3-amino-1-(*4*-methoxyphenyl)-4-(*2*-nitrophenyl) azetidin-2-one (4T). Brown solid (28%); Formula Weight: 313.31; MP 195 ºC;IR (KBr) 3282, 3064, 1745, 1509, 1243, 860 cm^-1^; ^1^H NMR (600 MHz, CDCl_3_) δ 7.17 – 7.13 (m, 6H), 7.09 (d, *J* = 7.7 Hz, 7H), 6.72 – 6.67 (m, 3H), 4.89 (s, 6H), 4.84 (d, *J* = 2.0 Hz, 3H), 4.55 (d, *J* = 2.0 Hz, 1H), 3.66 (s, 3H). Anal. calcd for: C_16_H_15_N_3_O_4_; Composition: C 61.3%, H 4.8%, N 13.4%.

(±)-*Trans*-3-amino-1-(*4*-methoxyphenyl)-4-(*p*-tolyl)azetidin-2-one (5T). Brown solid (95%); Formula Weight: 282.34; MP 110 ºC; IR (KBr) 3294, 2952, 1730, 1509, 1242, 824 cm^-1^; ^1^H NMR (600 MHz, CDCl_3_) δ 7.23 – 7.18 (m, 6H), 7.15 (m, 2H), 7.10 (d, *J* = 7.9 Hz, 13H), 7.06 (d, *J* = 8.1 Hz, 3H), 6.74 – 6.68 (m, 1H), 4.52 (d, *J* = 2.1 Hz, 1H), 3.93 (d, *J* = 2.1 Hz, 1H), 3.66 (s, 3H) 2.36 – 2.32 (m, 3H). Anal. calcd for: C_17_H_18_N_2_O_2_; Composition: C, 72.3%; H 6.4%; N, 9.9%.

(±)-*Trans*-3-amino-1-phenyl-4-(*p*-tolyl)azetidin-2-one (6T). Brown solid (62%); Formula Weight: 252.31; MP 135 ºC; IR (KBr) 3354, 3028, 1731, 1501, 792 cm^-1^; ^1^H NMR (600 MHz, CDCl_3_) δ 7.21 (dt, *J* = 11.6, 3.1 Hz, 2H), 7.18 – 7.14 (m, 4H), 7.11 (d, *J* = 7.9 Hz, 2H), 7.00 – 6.94 (m, 1H), 4.57 (d, *J* = 2.2 Hz, 1H), 3.96 (d, *J* = 2.3 Hz, 1H), 2.32 – 2.23 (m, 3H), 1.78 (s, 3H). Anal. calcd for: C_16_H_16_N_2_O; Composition: C, 76.2%; H, 6.4%; N, 11.1%.

(±)-*Trans*-3-amino-1-(4-methoxyphenyl)-4-(2-pyridyl)azetidin-2-one (7T). Brown solid (60%);Formula Weight: 269.30; MP 114 ºC;IR (KBr) 3371, 2953, 1729, 1510, 1294, 820 cm^-1^; ^1^H NMR (600 MHz, CDCl_3_) δ 8.71 (d, *J* = 4.0 Hz, 3H), 7.74 (td, *J* = 7.7, 1.6 Hz, 3H), 7.33 (d, *J* = 7.7 Hz, 4H), 7.31 – 7.27 (m, 13H), 6.82 (d, *J* = 9.1 Hz, 6H), 5.32 (d, *J* = 2.3 Hz, 3H), 4.76 (d, *J* = 2.3 Hz, 3H), 3.77 (s, 3H). Anal. calcd for: C_15_H_15_N_3_O_2_; Composition: C, 66.9%; H, 5.6%; N, 15.6%.

### Molecular docking

By means of computational chemistry, a molecular docking assay was performed targeting the protein β-tubulin. The drug-protein interaction of all the fourteen 3-amino-azetidin-2-one derivatives (1C–7C and 1T–7T) was studied on the three available and known binding sites (vinca, taxol and colchicine) of β-tubulin [17]. Vinblastine, taxol (paclitaxel) and colchicine were considered as the control molecules and the free energy (Kcal/mol) for each compound was calculated (Table 1).

**Figure 1:**
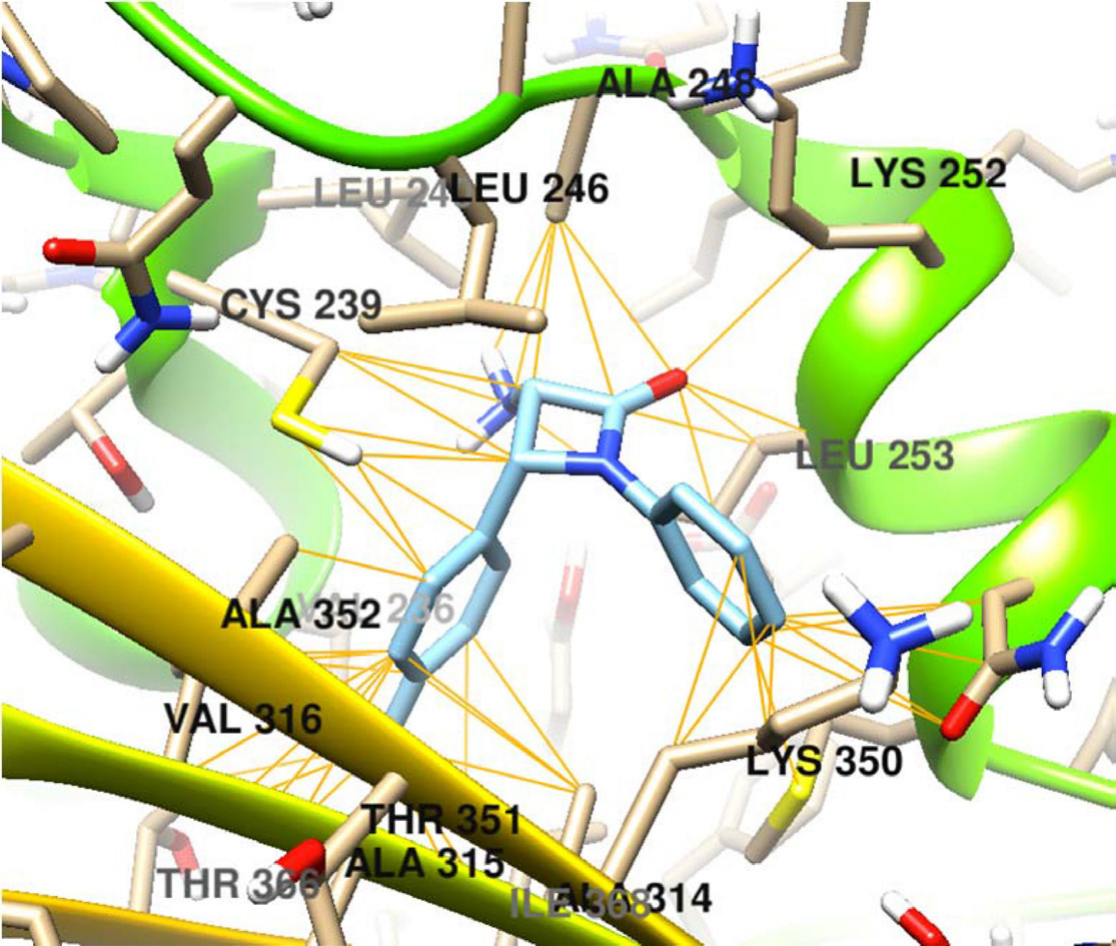
Molecular docking of the compound 6C on β-tubulin molecule

The docking interactions of (±)-*Cis*-3-amino-1-phenyl-4-(*p*-tolyl)azetidin-one (6C) is shown in Figure 1.

### Cytotoxic activity

The IC_50_ values of the fourteen 2-azetidinone derivatives (seven pair of diastereomers) were calculated from the concentrations of 10 to 0.312 μM for each compound (triplicate) against SiHa (human cervical), B16F10 (murine melanoma), K562 (human immortalized myelogenous leukemia) and Chang cell lines. IC_50_ values of the azetidin-2-ones are shown in Table 2.

**Table 2:** IC_50_ values (μM) of β-lactam derivatives against SiHa, B16F10, K562 and Chang cell lines (triplicate)

Compound code	SiHa	B16F10	K562	Chang
1T	7.17 ± 0.3	5.16 ± 0.5	> 10.00	7.91 ± 0.4
2T	7.14 ± 0.5	7.63 ± 0.4	> 10.00	> 10.00
3T	> 10.00	> 10.00	> 10.00	> 10.00
4T	8.12 ± 0.4	> 10.00	> 10.00	> 10.00
5T	7.79 ± 0.5	7.14 ± 0.6	> 10.00	> 10.00
6T	4.79 ± 0.6	5.79 ± 0.4	> 10.00	> 10.00
7T	> 10.00	> 10.00	> 10.00	> 10.00
1C	4.21 ± 0.4	3.33 ± 0.3	> 10.00	6.21 ± 0.5
2C	6.20 ± 0.6	1.71 ± 0.3	> 10.00	8.03 ± 0.5
3C	5.10 ± 0.4	3.91 ± 0.3	6.14 ± 0.4	9.32 ± 0.6
4C	7.28 ± 0.5	5.91 ± 0.6	> 10.00	7.37 ± 0.4
5C	4.03 ± 0.5	2.09 ± 0.1	> 10.00	7.01 ± 0.1
6C	2.34 ± 0.2	0.70 ± 0.1	> 10.00	6.89 ± 0.3
7C	8.43 ± 0.3	> 10.00	6.86 ± 0.4	5.49 ± 0.4
Colchicine	2.5 ± 0.5	4.1 ± 0.1	1.3 ± 0.1	0.52 ± 0.1
Paclitaxel	364 ± 310*	9.57 ± 0.2	4.80 ± 0.4*	0.068 ± 0.02
Vinblastine	10.88 ± 0.8	55 ± 28*	0.006 ± 0.0	ND**

* ng/mL; **Not determined.

**Figure 2:**
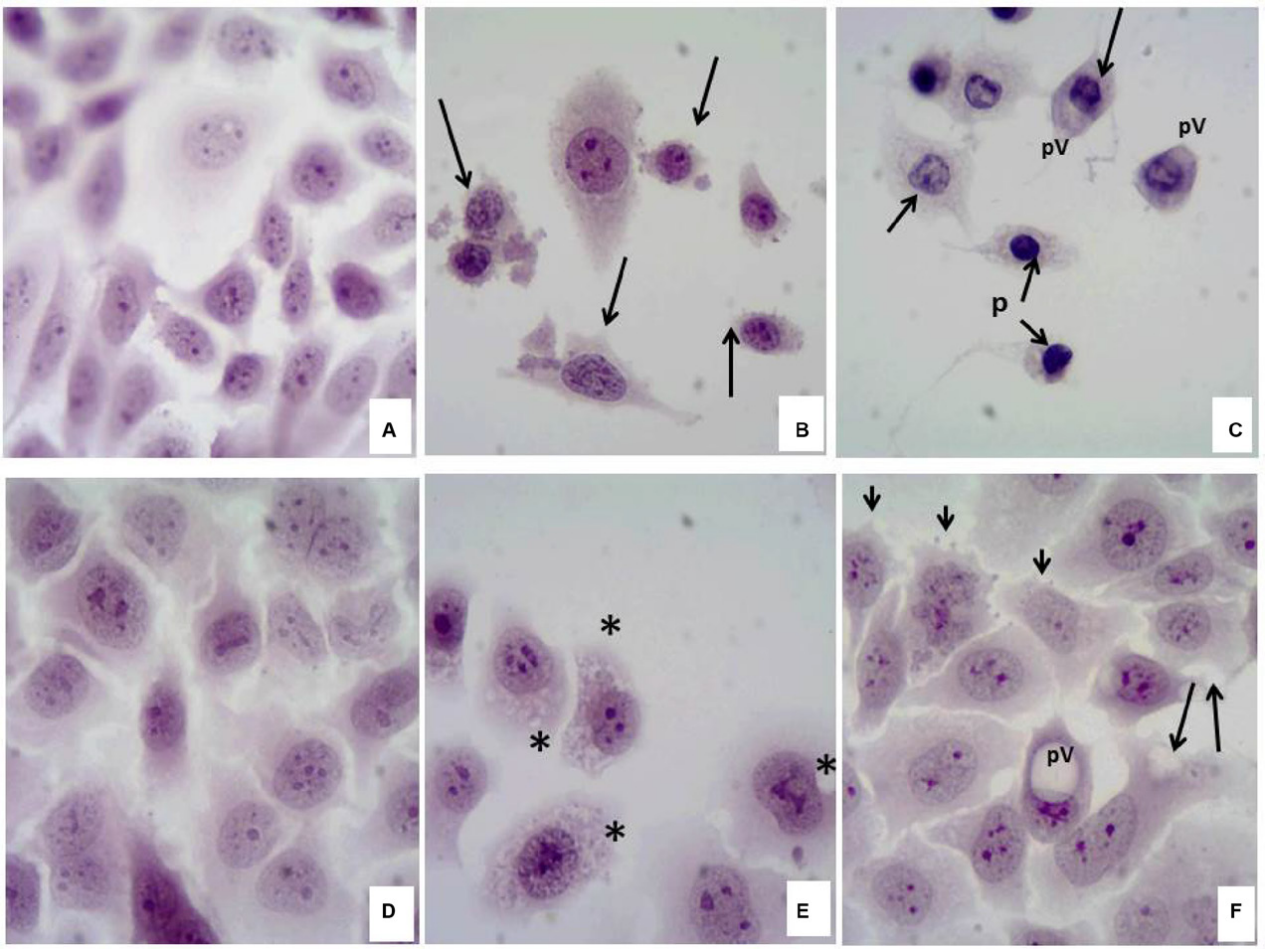
Cytotoxic effect of the compound 6C in SiHa and Chang cells

The cytotoxic effect of the compound 6C in SiHa and Chang cells has been studied (Figure 2). In a Hematoxylin/Eosin staining sample where monolayers of SiHa (a) and Chang (d) cells in the untreated control groups (negative control) showed the characteristic morphology of normal cell cultures, irregularly shaped, well-defined junctions and nucleus-cytoplasm ratio was preserved. In SiHa cells incubated with 1 μM of the compound 6C (b) morphological alterations was observed; mainly characterized by loss of cytoplasm and decrease in the size of nuclei (arrows). Moreover, in the incubated Chang cells, 1 μM concentration of the compound 6C (e), the damage could be characterized by the appearance of multiple intracytoplasmic vacuoles (*). Treatment with taxol (20 mg/mL) induced severe cell damage in SiHa cells (c), where nuclear membrane pucker (creaser) (n), perinuclear vacuoles (pv), loss of cytoplasm (arrows) and nuclear picnosis (p) were observed. On the other hand, Chang cells (f) experienced early apoptosis, was indicated by protrusions of the cell membrane (membrane blebbing) (arrowhead), cytoplasmic vacuoles (arrows) and sporadically large perinuclear vacuoles (pv). The morphological studies clearly demonstrated different susceptibility between SiHa and Chang cells to the identical treatment as well as different mechanism of cell damage for the investigative compound as well as taxol in SiHa and Chang cell lines although both the compounds (6C and taxol) ruptured the cellular monolayer.

Alternatively, the *trans* diastereomers exhibited moderate to poor (IC_50_: 4.79 to >10.00 in SiHa and 5.16 to > 10.00 in B16F10 cell lines) anticancer activity against the tested cancer cell lines as well as in Chang (normal liver) cells (Table 2).

### Apoptotic activity

**Figure 3:**
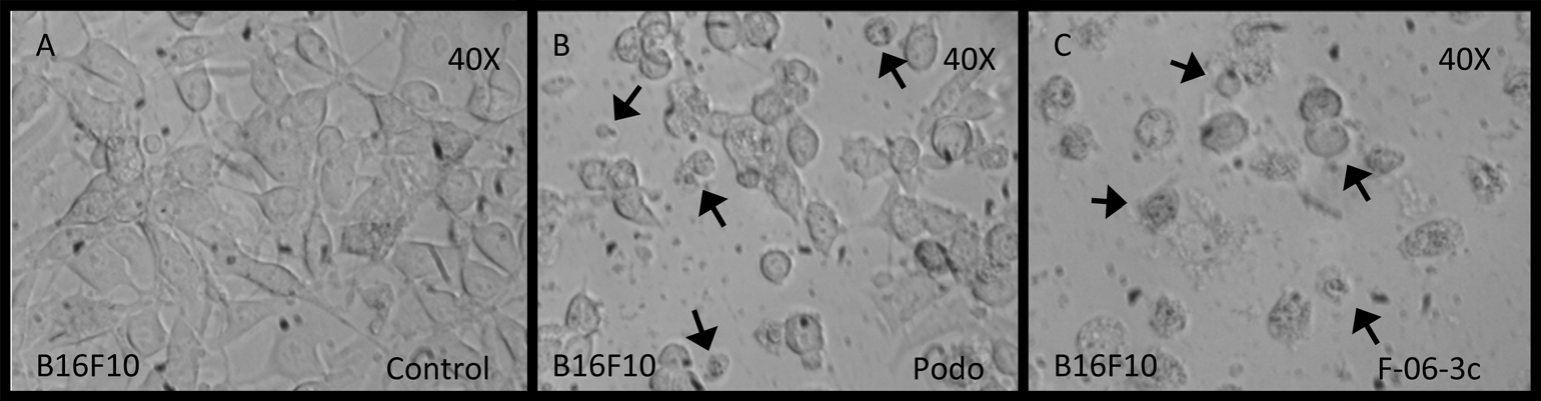
B16F10 cells under conditions of apoptosis with podophyllotoxin and 6C (**A**) Control untreated cells, (**B**) Cells treated with podophyllotoxin, (**C**) Cells treated with the compound 6C.

The *cis*-diastereomer 6C demonstrated apoptosis in B16F10 cancer cells through caspase-3 activation, was determined by choosing podophyllotoxin (a γ-lactone bearing pyrogallol derivative) as positive control (Figure 3).

## DISCUSSION

**Figure 4:**
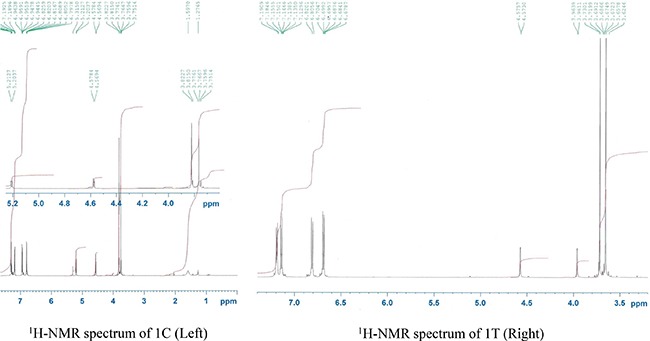
^1^H NMR spectra of 1C (Left) and 1T (Right)

It is always challenging to control the diastereoselectivity in β-lactam synthesis. The *cis* and *trans* diastereomers can be identified by the coupling constant values of the protons H-3 and H-4 in the β-lactam ring (*J*_3,4_ > 4.0 Hz represents a *cis* isomer whereas J_3,4_ < 3.0 Hz indicates *trans* stereoisomer) [18]. As a representative example the ^1^H NMR spectra of the compounds *(±)-trans*-3-amino-1,4-*bis*(*4*-methoxyphenyl)azetidin-2-one (1T) and (±)-cis-3-amino-1,4-*bis*(*4*-methoxyphenyl)azetidin-2-one (1C) could be considered (Figure 4). For the compound (1T) the two protons H-3 and H-4 appeared at 3.96 ppm (*J* = 1.7 Hz) and 4.57 ppm (*J* = 1.6 Hz) respectively whereas in the compound (1C), the coupling constant values for both the protons H-3 and H-4 are the same (*J* = 5.0 Hz). In fact, the diastereoselectivity in β-lactam formation depends on the generation of zwitterionic intermediate and the extent of conrotatory cyclization (ring-closure) of the intermediate to produce *cis* or *trans* β lactams [19, 20].

It is well-known that stereochemistry of a drug molecule is greatly responsible for its pharmacological activity. Accordingly, the stereoisomer that produces better therapeutic activity is known as eutomer and the less potent (sometimes inactive or even toxic) stereoisomer is known as distomer. The drugability of the newly synthesized terminal β-lactams (1C–1T and 1T–7T) has been validated by modified ‘*Rule of 5*’ (RO5) in Supplementary Table 1. It was found that all the fourteen newly synthesized β-lactams possess durglikeness and consequently can be termed as possible ‘*hits*’. The *in vitro* anticancer evaluation has been carried out against SiHa (human cervical carcinoma), B16F10 (murine melanoma), K562 (human immortalized myelogenous leukemia) and Chang cell lines. It has also been confirmed that the mechanism of anticancer activity was apoptosis-induced cell death. Finally, through a molecular docking analysis, a better and clearer understanding of the plausible β-lactam–β-tubulin interaction was explained, considering the protein β-tubulin as the therapeutic target.

The free energy coupling values for the *cis* and *trans* β-lactams on the proposed target β-tubulin do not match with that of two widely used natural anticancer drugs taxol and vincristine rather the values match with that of another anticancer alkaloid colchicine. This indicates that the binding sites of the β-lactams and colchicine are similar in nature. Accordingly, the detail mechanism of action of the β-lactams might be similar to that of colchicine. The docking score of the compound 6C is better than the natural drug colchicine (-7.6 compared to -7.4 for colchicine, Table 1) supports better anticancer activity of this compound than colchicine which has been confirmed by *in vitro* anticancer evaluation (Table 2). The IC_50_values of the compound 6C are better than colchicine against SiHa and B16F10 cancer cell lines. In fact, the drug-protein interactions depend on the chemical nature and three dimensional spatial orientation of the drug molecule and the subsequent interactions with the amino acid residues on the binding sites in β-tubulin. The molecular interactions of the compound 6C with β-tubulin showed bonding interactions with the Cys 239, Lys 252, Val 323, Met 257, Lys 350, Leu 246 and Ala 248 amino acid residues (Figure 1). Based on the accumulated data a strong interaction between the β-lactam pharmacophore and the aminoacid residues of the β-tubulin can be proposed.

Interestingly, the compound 6C showed strong *in vitro* anticancer activity (IC_50_ = 0.7 μM) in murine melanoma (B16F10) cell lines and IC_50_ = 2.34 μM in human cervical carcinoma SiHa cell lines and qualified as the lead with considerable anticancer activity in comparison to colchicine. The *trans*-β-lactams 1T, 2T, 4T, 5T, and the *cis*-β-lactams 2C, 3C, 4C and 7C showed good *in vitro* anticancer activity (IC_50_ = 5 – 10 μM) against SiHa cancer cell lines whereas the β-lactams 1T, 2T, 5T and 4C demonstrated good *in vitro* anticancer activity (IC_50_ = 5 –10 μM) against B16F10 cancer cell lines. On the other hand, two compounds 2C and 3C showed strong *in vitro* anticancer activity (IC_50_ < 5 μM) against B16F10 cancer cell lines. Besides the lead compound 6C, two other compounds 1C and 5C demonstrated strong *in vitro* anticancer activity (IC_50_ = 5–10 μM) against SiHa and B16F10 cancer cell lines. Interestingly, two *cis*-compounds 3C and 7C showed good *in vitro* anticancer activity (IC_50_ = 5–10 μM) against K562 cancer cell lines. All the fourteen compounds are less toxic (more selective) than the positive control colchicine, a well-known cancer chemotherapeutic agent. In Chang cells 6C showed 10 times lesser toxicity in comparison to the drug colchicine (Table 2).

## MATERIALS AND METHODS

### Chemistry

Melting points were determined in a Fisher Scientific electrochemical Mel-Temp* manual melting point apparatus (Model 1001) equipped with a 300ºC thermometer. FT-IR spectra were recorded on a Bruker Alpha modular Platinum-ATR FT-IR spectrometer with OPUS software, using the samples directly (neat) without making pallets. ^1^H NMR (600 MHz) and ^13^C NMR (150 MHz) spectra were obtained at room temperature with Bruker superconducting Ultrashield Plus 600 MHz NMR spectrometer with central field 14.09 T, coil inductance 89.1 H, and magnetic energy 1127.2 kJ using CDCl_3_ as solvent. All the solvents were purchased from Fisher-Scientific throughout the investigation. Dichloromethane and triethylamine were dried following the standard procedures. Deionized water was used for the preparation of all aqueous solutions.

### General procedure for the synthesis of imines

Amine and aldehyde were mixed in equimolar (1:1) ratio and refluxed in dry toluene using a Dean-Stark water separator. After completion of the reaction (monitored by thin layer chromatography, 2–3 hours), the solvent was removed under reduced pressure distillation (by rotavapor) and the pure imine was isolated by crystallization from dichloromethane/hexanes.

### General procedure for the synthesis of 3-phthalimido β-lactams *via* the Staudinger reaction

A representative experimental procedure is described as follows: A solution of phthalimidoacetic acid (*N*-phthaloylglycine) (1.5 mmol) in anhydrous dichloromethane (10 mL) was added (dropwise during 30 minutes) to another solution consisted of 2-chloro-1-methylpyridinium iodide (Mukaiyama reagent, 3 mmol) and triethylamine (6 mmol) in 10 mL dry dichloromethane at 0 to (–5)°C temperature under inert (argon) atmosphere. This mixture was stirred for 2 hours maintaining the same temperature. Imine (1 mmol) in dry dichloromethane (10 mL) was then added to this mixture at the same temperature. The reaction mixture was then stirred overnight at room temperature and monitored by thin layer chromatography (TLC). After completion of the reaction, the reaction mixture was washed with saturated sodium bicarbonate solution (10 mL), brine (10 mL) and deionized water successively. The organic layer was dried over anhydrous sodium sulfate, filtered and evaporated to obtain the crude product. Column chromatography over silica gel was performed using ethyl acetate/hexanes mixtures to isolate the *cis*- and *trans*-3-phthalimido β-lactams in pure form. The *cis:trans* ratio was approximately 2:1 in all the cases.

### General procedure for the synthesis of 3-amino β-lactams from 3-phthalimido β-lactam

Ethylenediamine (2 mmol) was added to a solution of diastereomerically pure 3-phthalimido β-lactam (1 mmol) in anhydrous ethyl alcohol (5 mL) under argon atmosphere. This mixture was stirred at room temperature and the progress of the reaction was monitored by TLC. After completion of the reaction (15 min–1 h) the ethanol was removed by reduced pressure distillation and the crude mass was extracted with ethyl acetate (25 mL). The ethyl acetate layer was washed with brine and water successively (10 mL), and dried over anhydrous sodium sulfate. The pure products were obtained *via* column chromatography over silica gel using ethyl acetate/hexanes as the eluent.

### Molecular docking

One of the major cancer related proteins, β-tubulin, was considered as the theoretical target to perform docking studies of the azetidin-2-ones (β-lactams) as possible antiproliferative compounds. The hydrogens and charges on receptor and ligand were assigned with the Chimera program [21]. The pdbqt files for docking simulations were generated using the AutoDock Tools interface. The docking of azetidine-2-one derivatives on β-tubulin was performed with Autodock 4.2 program [22]. The grid sizes in each dimension (x, y, and z) were 60 Å, grid points separated by 0.375 Å, with its center in 116, 89 and 6 for x, y and z, respectively. The analysis of AutoDock predicted molecular interactions was conducted by AutoDock vina interface and Chimera program. Maestro software showed the key amino acids from colchicine binding site of β-tubulin.

### Biology

### Cytotoxic activity

*In vitro* anticancer assays were performed in triplicate for the determination of mean values. The human cervical *carcinoma* SiHa, murine melanoma B16F10, chronic myelogenous leukemia (CML) K562, and Chang cell lines were cultured in minimum essential medium of Eagle at 37 °C in a 95% O_2_/5% CO_2_ atmosphere with 10% fetal bovine serum, 2 mM L-glutamine and 1% penicillin/streptomycin. Cells were trypsinized and seeded at a density of 5 × 10^4^ cells/mL in a 96-well plate and incubated at 37 °C, 95% O_2_/5% CO_2_ atmosphere for 24 h. Thereafter the cells were treated with azetidin-2-one derivatives (dissolved in DMSO) at various concentrations between 312 nM–10 μM, and re-incubated for further 72 h. Wells containing the equal volume of the vehicle DMSO (1% v/v) were used as background (negative control). The culture medium was then removed and the cells washed with 100 μL phosphate buffered saline (PBS); then 10 μL WST-1 (Roche, USA) was added. Cells were incubated for 2 h in absence of light at 37°C, and absorbance was read subsequently [23]. The absorbance value of cells without added compound was set to 100% cell viability and was compared versus cell death density (cells treated with Triton ×100) and also natural anticancer drug colchicine as positive control compound. Afterwards, these values were used to assess half maximal inhibitory concentration (IC_50_) using Graph-Pad Prism software.

### Hematoxylin/Eosin staining of the cells

Cells (10 × 10^4^) were grown on coated poly-lysine coverslips which were placed in 6-well microplates overnight and then incubated for 24 h in the presence of compound 6C or taxol, subsequently stained with hematoxylin and eosin. Coverslips were then observed by Light microscopy of bright field (40X).

### Apoptotic activity

The mechanism of cell death was identified as apoptosis. It was validated with the compound 6C. Enzyme activity associated with the induction of apoptosis was determined by caspase-3 assay (Invitrogen, USA) as described in the protocol [24]. A concentration of approximately 3 × 10^6^ cells/mL (B16F10) was taken and incubated for 24 h at 37 °C in 5% CO_2_ environment. Podophyllotoxin (apoptosis inducing agent) was used as positive control. After incubation for 24 h the cells were re-suspended and 50 μL of cell lysis reagent was added, then cells were incubated for 10 min in ice bath and centrifuged. The supernatant (cytosol extract) was transferred into a new effendrof. Cytosol extract was diluted with cell lysis reagent to a concentration of 2 μg/μL, in a total volume of 50 μL. Then 50 μL of 2X reaction buffer and 5 μL of tetrapeptide-*p*-nitroaniline (DEVD-*p*NA) were added. It was incubated at 37 °C for 2 h (in dark), and finally the optical density at 405 nm was measured with an ELISA Reader [24]. Caspase-3 activity was calculated that confirmed apoptosis as the mechanism of cell death. With this data and the value of caspasae-3 induction by the podophyllotoxin, it was possible to calculate the ratio of apoptosis with respect to the positive control on murine melanoma cancer cells (B16F10).

## CONCLUSIONS

In conclusion, a series of fourteen (seven pairs of *cis-* and *trans-*diastereomers) diversely substituted diastereomeric β-lactam derivatives have been successfully synthesized. *In vitro* anticancer evaluation showed that one of these newly synthesized *cis*-β-lactam derivatives (6C) possesses higher potency in human cervical carcinoma SiHa cell lines, six times higher potency in murine melanoma B16F10 cell lines and 10 times less toxicity in normal liver Chang cell lines compared to the positive control colchicine, a well-known anticancer alkaloid. Besides the cis-β-lactam 6C, most of the newly synthesized β-lactams (both diastereomers) exhibited moderate to strong anticancer activity against SiHa and B16F10 cancer cell lines. The binding sites of the compound on β-tubulin was found to be similar with that of colchicine and apoptotic cell death through caspase activation was found as the mechanism of action. It was supported by the microscopic observation. Molecular coupling of the compound 6C on β-tubulin colchicine site, exhibited binding interactions with the Cys 239, Lys 252, Val 323, Met 257, Lys 350, Leu 246 y Ala 248 amionacid residues by molecular docking assay. The molecule 6C may find its application in anticancer drug discovery process.

### Dedication statement

A heartfelt tribute to the memory of Professor Asima Chatterjee, the legendary scientist-teacher-humanitarian, on the occasion of her birth centenary (1917–2017).

## SUPPLEMENTARY MATERIALS TABLES AND FIGURES




