# Supplementary material for: Stereochemical preference toward oncotarget: Design, synthesis and in vitro anticancer evaluation of diastereomeric β-lactams
Source: Oncotarget. 2017 May 22;8(23):37773–82. doi: 10.18632/oncotarget.18077 (PMC5514948; doi:10.18632/oncotarget.18077)

Stereochemical preference toward oncotarget: Design, synthesis and *in vitro* anticancer evaluation of diastereomeric β-lactams

Supplementary Materials

Supplementary Table 1: Structure and druggability validation† of the fourteen (seven pairs) of diastereomeric β-lactam derivatives. Colchicine was used as the positive control.
See Supplementary_Table_1

Graphical abstract

Comparative anticancer activity (*in silico* and *in vitro*) of the *cis*- and *trans*- β-lactams has been studied. The compound (±)-*Cis*-3-amino-1-phenyl-4-(*p*-tolyl) azetidin-2-one was found to be more active and site selective than the marketed anticancer drug colchicine.

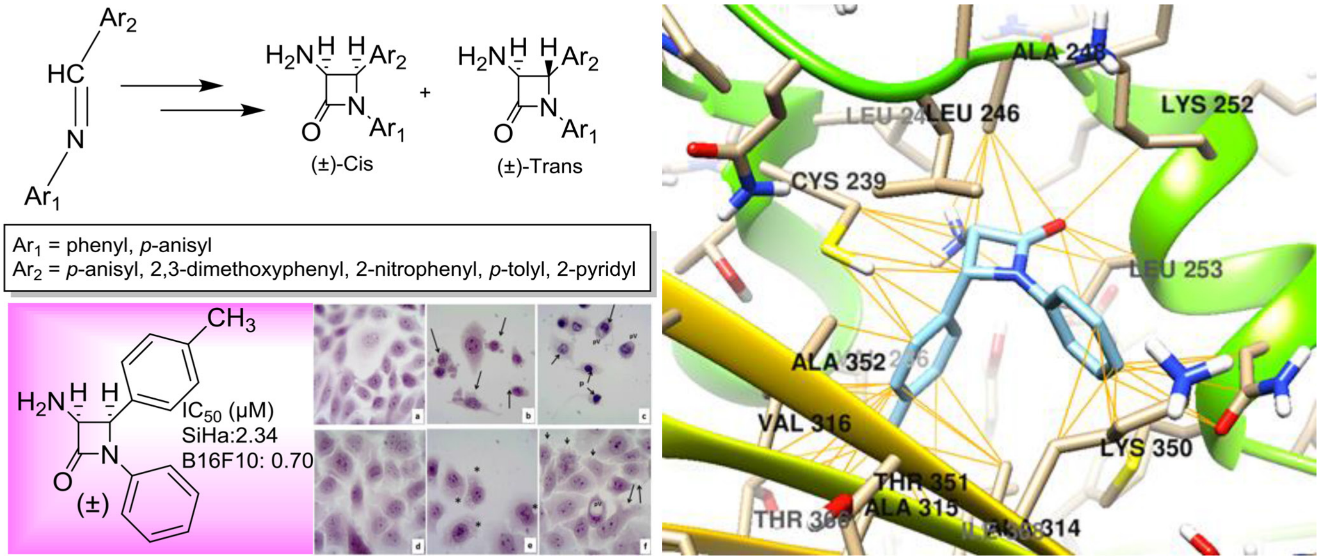

Supplement: Supplementary file 1 [file oncotarget-08-37773-s001.pdf]
