# Supplementary material for: Stereochemical preference toward oncotarget: Design, synthesis and in vitro anticancer evaluation of diastereomeric β-lactams
Source: Oncotarget. 2017 May 22;8(23):37773–82. doi: 10.18632/oncotarget.18077 (PMC5514948; doi:10.18632/oncotarget.18077)
Supplement: Supplementary file 2 [file oncotarget-08-37773-s002.docx]

**Supplementary Table 1:** Structure and druggability validation^†^ of the fourteen (seven pairs) of diastereomeric β-lactam derivatives. Colchicine was used as the positive control

| **Code** | **Structure** | **miLogP^a^** | **HBA^b^** | **HBD^c^** | **TPSA^d^** | **RB^e^** | **MW^f^** |
| --- | --- | --- | --- | --- | --- | --- | --- |
| 1C |  | 1.78 | 5 | 2 | 64.80 | 4 | 298 |
| 1T |  | 1.78 | 5 | 2 | 64.80 | 4 | 298 |
| 2C |  | 1.72 | 4 | 2 | 55.57 | 3 | 268 |
| 2T |  | 1.72 | 4 | 2 | 55.57 | 3 | 268 |
| 3C |  | 1.37 | 6 | 2 | 74.03 | 5 | 328 |
| 3T |  | 1.34 | 6 | 2 | 74.03 | 5 | 328 |
| 4C |  | 1.64 | 7 | 2 | 101.39 | 4 | 313 |
| 4T |  | 1.63 | 7 | 2 | 101.39 | 4 | 313 |
| 5C |  | 2.17 | 4 | 2 | 55.57 | 3 | 282 |
| 5T |  | 2.08 | 4 | 2 | 55.57 | 3 | 282 |
| 6C |  | 2.12 | 3 | 2 | 46.33 | 2 | 252 |
| 6T |  | 2.21 | 3 | 2 | 46.33 | 2 | 252 |
| 7C |  | 0.55 | 5 | 2 | 68.46 | 3 | 269 |
| 7T |  | 0.55 | 5 | 2 | 68.46 | 3 | 269 |
| Colchicine  (positive  control) |  | 1.39 | 7 | 1 | 83.81 | 6 | 431 |

^†^Molinspiration property engine v2016.10

^a^miLogP: Moriguchi octanol-water partition coefficient, is based on quantitative structure-LogP relationships, by using topological indexes

^b^Hydrogen bond acceptor

^c^Hydrogen bond donor

^d^Total polar surface area

^e^Number of rotatable bonds
